# Exacerbations of Chronic Rhinosinusitis—Microbiology and Perspectives of Phage Therapy

**DOI:** 10.3390/antibiotics8040175

**Published:** 2019-10-05

**Authors:** Joanna Szaleniec, Agnieszka Gibała, Monika Pobiega, Sylwia Parasion, Jacek Składzień, Paweł Stręk, Tomasz Gosiewski, Maciej Szaleniec

**Affiliations:** 1 Department of Otolaryngology, Faculty of Medicine, Jagiellonian University Medical College, Sniadeckich 2, 31-531 Krakow, Poland; jacek.skladzien@uj.edu.pl (J.S.); pawel.strek@uj.edu.pl (P.S.); 2 Jerzy Haber Institute of Catalysis and Surface Chemistry Polish Academy of Sciences, Niezapominajek 8, 30-239 Krakow, Poland; gibala.agnieszka@gmail.com (A.G.); ncszalen@cyfronet.pl (M.S.); 3 Maria Sklodowska-Curie Memorial Cancer Center and Institute of Oncology, Cracow Branch, Garncarska 11, 31-115 Krakow, Poland; 4 Biophage Pharma S.A., Mogilska 40, 31-546 Krakow, Poland; monika.pobiega@gmail.com (M.P.); sylwia.parasion@biophage.pl (S.P.); 5 Department of Molecular Medical Microbiology Unit, Faculty of Medicine, Jagiellonian University Medical College, Czysta 18, 31-121 Krakow, Poland; tomasz.gosiewski@uj.edu.pl

**Keywords:** rhinosinusitis, exacerbations, bacteriophage, phage, antibiotic resistance, biofilm

## Abstract

The chronically inflamed mucosa in patients with chronic rhinosinusitis (CRS) can additionally be infected by bacteria, which results in an acute exacerbation of the disease (AECRS). Currently, AECRS is universally treated with antibiotics following the guidelines for acute bacterial rhinosinusitis (ABRS), as our understanding of its microbiology is insufficient to establish specific treatment recommendations. Unfortunately, antibiotics frequently fail to control the symptoms of AECRS due to biofilm formation, disruption of the natural microbiota, and arising antibiotic resistance. These issues can potentially be addressed by phage therapy. In this study, the endoscopically-guided cultures were postoperatively obtained from 50 patients in order to explore the microbiology of AECRS, evaluate options for antibiotic treatment, and, most importantly, assess a possibility of efficient phage therapy. *Staphylococcus aureus* and coagulase-negative staphylococci were the most frequently isolated bacteria, followed by *Haemophilus influenzae, Pseudomonas aeruginosa,* and *Enterobacteriaceae*. Alarmingly, mechanisms of antibiotic resistance were detected in the isolates from 46% of the patients. Bacteria not sensitive to amoxicillin were carried by 28% of the patients. The lowest rates of resistance were noted for fluoroquinolones and aminoglycosides. Fortunately, 60% of the patients carried bacterial strains that were sensitive to bacteriophages from the Biophage Pharma collection and 81% of the antibiotic-resistant strains turned out to be sensitive to bacteriophages. The results showed that microbiology of AECRS is distinct from ABRS and amoxicillin should not be the antibiotic of first choice. Currently available bacteriophages could be used instead of antibiotics or as an adjunct to antibiotics in the majority of patients with AECRS.

## 1. Introduction

According to recent studies, at least 1 in 10 citizens of Europe and the USA struggles with chronic rhinosinusitis (CRS) [1,2]. Persistent symptoms that define the disease, such as nasal congestion and discharge, facial pain, and loss of smell, can be even more debilitating than serious cardiovascular or pulmonary problems [3]. Billions of dollars are spent yearly on antimicrobials for patients with sinusitis and research on the subject [4,5], but surprisingly, it seems that many essential questions concerning the actual role of bacteria in CRS still remain unanswered. In this article, we discuss the indications for antibacterial treatment in patients with CRS and investigate whether they could benefit from bacteriophage therapy.

### 1.1. Antimicrobial Treatment in Patients with CRS

CRS is a multifactorial inflammatory disorder and currently it is no longer regarded as a primarily infectious process [6]. Nevertheless, antibiotics are frequently prescribed for patients with CRS [7]. The rationales behind this line of treatment are as follows:

(1) Antibiotics are expected to alleviate the baseline symptoms of CRS, because they decrease the load of bacteria that may supposedly play a role in perpetuating the inflammation [6];
(2) Antibiotics (or other novel antimicrobials) are hoped to reduce bacterial biofilms that contribute to the recalcitrance of CRS [8,9,10];
(3) Antibiotics eliminate bacteria that cause acute exacerbations of CRS (AECRS) i.e., acute infections that temporarily worsen the chronic symptoms.

As discussed below, the indications for antimicrobial therapy are best established in AECRS, while the evidence supporting its use in the first two clinical situations is ambiguous.

(1) CRS (baseline symptoms).

The role of bacteria in the etiology of CRS is poorly understood [11]. Current studies have shown that diverse microbial communities exist in the sinuses both in healthy subjects and in patients with sinusitis [12,13]. The cause–effect relationship between the presence of bacteria and the symptoms of CRS is usually far from apparent [14]. In fact, the etiology of CRS is very complex. In many patients the symptoms can be caused by multiple other factors and the microorganisms dwelling in the sinuses may have virtually no impact on the basic course of the disease. Therefore, therapy directed against these bacteria does not result in clinical improvement [15].

It is possible that in the near future antimicrobials will find their place in the treatment of CRS. Current understanding of sinonasal dysbiosis and its significance for the pathogenesis of the disease is insufficient to introduce any rational antimicrobial therapy that could restore a beneficial microbiota.

(2) Biofilms.

Bacterial biofilms are associated with the most severe forms of rhinosinusitis [8,9,10]. Consortia of microorganisms embedded in an extracellular matrix are 1000-fold more resistant to antibiotics than planktonic bacteria [6]. Unfortunately, despite vigorous research, currently available antimicrobials are unable to induce sufficient eradication of the sinonasal biofilms [16].

(3) Bacterial exacerbations of CRS (AECRS).

The causative role of bacteria seems to be most apparent in infectious exacerbations of CRS. AECRS is defined as a sudden worsening of symptoms with a return to baseline symptoms after treatment [17,18,19]. Exacerbations can be caused by bacteria or other factors (allergy, virus, etc.). During an acute bacterial exacerbation, the aggravation of symptoms occurs when the chronically inflamed mucosa is additionally temporarily invaded by bacteria. Bacterial etiology is usually suspected if there is evidence of purulent secretions on nasal endoscopy [20,21]. Most experts agree that acute bacterial exacerbations of CRS require antimicrobial treatment [6,22,23,24]. It is important to note that in this case antimicrobials are not expected to *cure* CRS, but rather to clear out the symptoms of exacerbation and help the patient to return to their “baseline severity” of the disease.

In the light of current evidence, patients with CRS are most likely to benefit from any kind of culture-directed antimicrobial treatment when they present with signs of bacterial exacerbations. For this reason, we chose this particular group of patients to investigate the possibility of introducing phage therapy as an alternative for traditional antibiotic therapy.

### 1.2. Bacteriophage Therapy Versus Antibiotic Therapy for AECRS

In clinical practice, antibiotic therapy of AECRS frequently proves ineffective or the patients experience rapid recurrence of the infection. The disease can be recalcitrant to antibiotic treatment for several reasons [6,11,24]:

- Increasing prevalence of antibiotic-resistant bacteria is observed in sinonasal infections worldwide;
- Biofilms that constitute a bacterial reservoir for recurrent exacerbations prove virtually impossible to eradicate with antibiotics;
- Non-selective elimination of both pathogenic and potentially beneficial bacteria caused by antibiotics results in uncontrolled repopulation of the empty niches. This process cannot be controlled and may not lead to restoration of an ‘optimal’ microbial community. The role of potential probiotics is still too poorly understood to prevent reintroduction of pathogenic species;
- Repeated courses of antibiotic therapy may contribute to increasing antibiotic resistance of the patient’s microbiota;
- In some patients, antibiotics cause serious adverse effects or allergic reactions.

Bacteriophage therapy can potentially address the problems mentioned above. The bacteriophages (or phages) are viruses that infect and destroy bacterial cells. They have been used in the treatment of human infections for a hundred years now, however, in Western countries, they were all but forgotten after the introduction of antibiotics [25,26].

The bacteriophages have several advantages compared to antibiotics [27,28]:

- The mechanisms of antibiotic and phage resistance are entirely different. Therefore, bacterial strains that acquired antibiotic resistance frequently remain sensitive to phages;
- Some phages are able to penetrate and disrupt bacterial biofilms;
- The phages are highly selective. They eliminate only selected bacterial strains and leave the rest of the microbial community intact;
- Introduction of phage therapy instead of repeated antibiotic courses may prevent further selection of antibiotic-resistant strains;
- Phage preparations were shown to be generally safe and well-tolerated.

### 1.3. Aims of the Study

In this study, we decided to address several unclear issues regarding the microbiology of AECRS, such as species diversity and prevalence of antibiotic resistance among the strains isolated from AECRS patients. The second question concerns the activity of bacteriophages from the Biophage Pharma collection against the isolated bacteria, especially antibiotic-resistant strains, to evaluate the possibility of including phage therapy in the treatment of patients with AECRS in the future.

## 2. Results

Fifty patients with AECRS were included in the study. All of the patients had undergone endoscopic sinus surgery (ESS). The demographic and clinical characteristics of the study group are listed in Table 1. The majority of patients who presented with sinonasal infections to the outpatient clinic of the Department of Otolaryngology, Jagiellonian University Medical College in Krakow, had CRS with nasal polyps. Most of them had suffered from rhinosinusitis for many years and reported a long history of topical, and frequently also systemic, steroid treatment. Many had experienced recurrent exacerbations that required repeated antibiotic courses.

**Figure 1:**
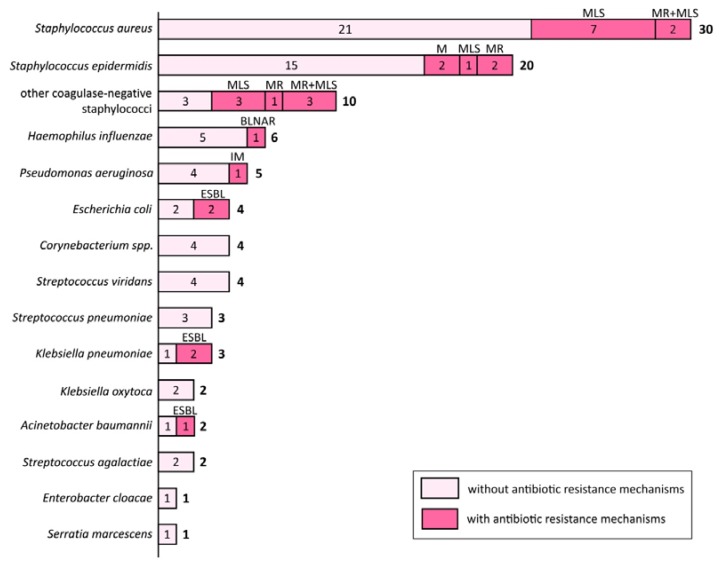
Bacteria cultured from the patients (number of isolates). In this figure we report all of the isolated species even if their pathogenicity remains controversial. Mechanisms of antibiotic resistance: MR—methicillin resistance, MLS—macrolide–lincosamide–streptogramin B resistance, ESBL—extended spectrum β-lactamases, M—M-phenotype (resistance to erythromycin), IM—reduced sensitivity to imipenem, BLNAR—β-lactamase negative ampicillin resistance.

A total of 97 isolates were recovered from the patients. A detailed list of the isolated species including information about their antibiotic-resistance mechanisms is shown in Figure 1. 

Mechanisms of antibiotic resistance were identified in 28% of the isolates. However, most patients harbored several strains (1–4, median 2 per patient), frequently both antibiotic-sensitive and antibiotic-resistant. Consequently, antibiotic-resistant bacteria were carried by 46% of patients. If both natural and acquired resistance were taken into account, 18% of the isolated strains obtained from 28% patients were not sensitive to amoxicillin/clavulanate. Even greater rates of resistance were observed for macrolides (25% of strains, 42% of patients) and clindamycin (30% of strains, 40% of patients). On the other hand, resistance to fluoroquinolones was very uncommon (6% of strains, 10% of patients). Similar results were noted for aminoglycosides (resistance to gentamicin in 4% of strains and 8% of patients). Reduced sensitivity to carbapenems was detected only in one strain of *Pseudomonas aeruginosa*. All of the isolates, including methicillin-resistant *Streptococcus aureus* (MRSA), were sensitive to linezolid.

Analysis of the distribution of bacteria in patients with various comorbidities proved that antibiotic-resistant strains were more frequently isolated from individuals with aspirin-exacerbated respiratory disease (AERD). In these patients, 53% of the isolated bacteria had antibiotic-resistance mechanisms compared to 25% in patients without AERD. The difference was statistically significant (chi-square 5.18, *p* = 0.02). Similar results were noted for patients with asthma (38% of isolates with resistance mechanisms versus 17% in patients without asthma, chi-square 5.09, *p* = 0.02). Also, the participants who reported recurrent exacerbations more frequently carried resistant strains, however, this trend did not reach statistical significance. The presence of other comorbidities did not correlate with any differences in the distribution of bacterial species.

Only in five (10%) patients a pathogen characteristic for acute rhinosinusitis (i.e., *Haemophilus influenzae* or *Streptococcus pneumoniae*) was the only one identified in the swab. In eight (16%) individuals we found solely bacteria of disputable pathogenicity, such as *Staphylococcus epidermidis* and other coagulase-negative staphylococci, *Streptococcus viridans* or *Corynebacterium* spp.

**Figure 2:**
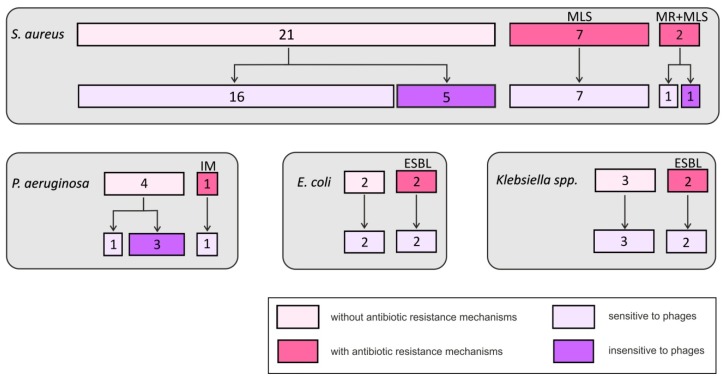
Sensitivity of *S. aureus, P. aeruginosa, E. coli,* and *Klebsiella* spp. to antibiotics and bacteriophages.

Phage typing was performed for *Staphylococcus aureus, Pseudomonas aeruginosa, Escherichia coli, Klebsiella pneumoniae, Klebsiella oxytoca*, and *Acinetobacter baumannii*. The vast majority (80%) of these isolates, including antibiotic-resistant strains, were sensitive to the bacteriophages from the collection used in the study, as shown in Figure 2. Bacteriophages for other bacterial species were not available in the collection. Bacteria that are traditionally not considered as pathogenic, such as coagulase-negative staphylococci, corynebacteria, and *S. viridans*, were not included in phage typing and further analyses. The remaining bacteria are further briefly referred to as “pathogens”.

**Figure 3:**
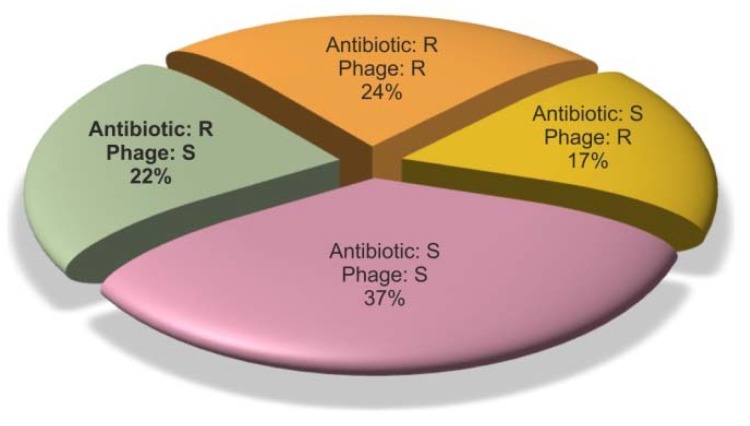
Comparison of phage and antibiotic sensitivity in the strains considered as “pathogens” (in this figure the species of disputable pathogenicity were excluded). S—sensitive, R—resistant.

Figure 3 compares the sensitivity of the pathogens isolated from the study group to antibiotics and bacteriophages. The strains were described as ‘antibiotic-resistant’ if they had mechanisms of antibiotic resistance and as ‘antibiotic-sensitive’ if no such mechanisms were identified. On the other hand, although the strains labeled as ‘phage-insensitive’ were not lysed by the phages from our collection, it does not necessarily mean that they are resistant to phages in general. Nevertheless, 59% of the pathogens, including 81% of the antibiotic-resistant pathogens, showed sensitivity to bacteriophages. Phage cocktails proved to be effective against 63% of *S. aureus* isolates and 40% of *P. aeruginosa* isolates.

As explained above, due to uneven distribution of the bacteria among patients, the prevalence of phage-sensitive bacteria in the study group needs to be presented separately from the analysis of phage sensitivity in the bacterial isolates. Nineteen (38%) patients carried only phage-sensitive pathogens, 11 (22%) had both phage-sensitive and phage-resistant pathogens, and 12 (24%) had only phage-insensitive pathogens (as mentioned above, the remaining 8 patients had only strains of disputable pathogenicity). The phage cocktails were effective in 63% of patients with *S. aureus* and 40% of patients with *P. aeruginosa.*

## 3. Discussion

### 3.1. Microbiology and Antibiotic Resistance in AECRS

The question how to treat AECRS is frequently encountered in clinical practice, but surprisingly seldom addressed in research. Currently, there are no evidence-based guidelines for the management of AECRS [17]. It is still a point of debate whether AECRS should be treated similarly to acute bacterial rhinosinusitis in patients without underlying chronic sinonasal disease (ABRS). In ABRS, the usual pathogens are *S. pneumoniae*, *H. influenzae*, and *Moraxella catarrhalis,* and the most commonly recommended antibiotics are amoxicillin or amoxicillin/clavulanate [31]. Meanwhile, the bacteriology of AECRS is far from clear. Some authors suggest that the microbiology of ABRS and AECRS can be similar. In two studies by Brook et al., *S. pneumoniae, H. influenzae,* and *M. catarrhalis* were the most commonly isolated aerobic bacteria in patients with AECRS [18,32]. However, many researchers share the opinion that the sinonasal microenvironment altered by CRS may impact the evolution of bacterial infections. Further changes in the microbiome composition and increased antibiotic resistance are observed after surgery and repeated antibiotic courses. Therefore, patients with AECRS (particularly after surgery) are likely to harbor different bacterial species and require different therapy than recommended for ABRS [17,33].

Our observations strongly support the assumption that the bacteriology of AECRS is distinct from ABRS. In our study, the species identified in more than a half of the isolates were *S. aureus*, *S. epidermidis,* and other coagulase-negative staphylococci, followed by *H. influenzae, P. aeruginosa,* and less commonly, *E. coli*, *S. pneumoniae, K. pneumoniae,* and *K. oxytoca.* Overall, the “acute pathogens” *S. pneumoniae* and *H. influenzae* constituted only 9% of the isolates. It is important to note that barely in five patients the “acute pathogen” was the only one isolated from the sinuses. In most cases it was accompanied by *S. aureus* or *P. aeruginosa.* The significance and pathogenic potential of coagulase-negative staphylococci in patients with AECRS remains unclear. In our patients, these species were also usually isolated together with other bacteria, and only in eight patients no other pathogenic species were identified.

Other authors who investigated populations similar to our study group (mainly post-ESS patients with correctly diagnosed AECRS) reported comparable findings [34,35,36,37,38]. The most commonly isolated pathogens were always *S. aureus* (25%–70%) and *P. aeruginosa* (9%–24%), while the “acute pathogens” *S. pneumoniae, H. influenzae,* and *M. catarrhalis* constituted only 10%–22% of all isolates. *S. epidermidis* was also frequently identified in the isolates (however, some authors considered it nonpathogenic and did not include it in their calculations). These results suggest that our observations can be considered as representative for the general population of patients with postoperative exacerbations of CRS.

Antibiotic-resistant bacteria are frequently isolated from patients with CRS [34,39]. In our study resistant strains were identified in almost half of the patients. The antibiotic sensitivity analysis indicated that amoxicillin should not be chosen as the first line or empiric treatment for post-ESS patients with AECRS, because they are likely to harbor strains not sensitive to amoxicillin. This finding stands in contrast to the recommendations for ABRS [22]. Fluoroquinolones and aminoglycosides provided the best chance for clinical success in our study group, but they need to be administered with caution due to their considerable toxicity. Therapeutic decisions in patients with AECRS are, therefore, not trivial. The patient’s individual contraindications and local patterns of resistance (primarily for *S. aureus* and *P. aeruginosa*) should be considered to choose optimal empiric therapy.

The most probable cause of antibiotic resistance in the patient’s microbiota is repeated antibiotic therapy in the past. It is not possible to assess retrospectively the number of antibiotic courses that the patients have received in their life. In our group, there was higher incidence of antibiotic-resistant strains in patients with self-reported recurrent exacerbations treated with antibiotics, but this result was not statistically significant. In fact, it is the patients with asthma and particularly AERD who typically present with the most severe and recalcitrant CRS and receive the most aggressive treatment, including repeated antibiotic courses. Indeed, as expected, the rates of antibiotic resistance in patients with asthma and AERD were extremely high. The patients’ medical history seems to be reflected in their microbiota, which is another argument to support the postulate to avoid the use of antibiotics unless it is truly necessary.

Many patients in our study group carried bacterial species such as coagulase-negative staphylococci that are traditionally considered as non-pathogenic but had mechanisms of antibiotic resistance. It is disputable whether these species can cause symptoms of exacerbations in patients with CRS or if they should be targeted by treatment. Nevertheless, even if they only constitute a part of the commensal flora, they may transfer the genes of antibiotic resistance to actual pathogens and thus reduce the clinical effectiveness of antibiotic therapy.

### 3.2. Phage Therapy for AECRS

Increasing antibiotic resistance is one of the most important triggers to search for alternative antimicrobials. Phage therapy is one of the most promising methods that frequently proved to be safe and effective when antibiotic treatment failed [25,27]. Nowadays, after many decades when it was almost forgotten in Western countries, the use of bacteriophages is gaining rising interest as a method that could solve many problems associated with antibiotic treatment.

Phage therapy has not yet been approved for use in exacerbations of CRS, but the results of preliminary studies and the first clinical trials are very promising [40]. Drilling et al. tested the in vitro efficacy of a bacteriophage cocktail against *S. aureus* strains isolated from Australian patients with CRS [41]. The cocktail lysed 94% (62/66) of the isolates in planktonic forms. Furthermore, the cocktail caused a reduction in biofilm mass for four out of five of the isolates. Similarly, Fong et al. proved that a cocktail of four phages was able to lyse 89% (40/45) of *P. aeruginosa* isolates from CRS patients from Australia, Europe, and the USA. Again, the cocktail significantly reduced *P. aeruginosa* biofilms grown in vitro [42]. Finally, Zhang et al. found that phage susceptibility of *S. aureus* isolates from CRS patients was not decreased in the most feared multidrug-resistant pathogens [43].

The safety and efficacy of the bacteriophages or their enzymes for sinonasal infections was shown in vivo in murine and ovine models [44,45,46,47,48]. For over a century, phage therapy has also been used in humans without significant adverse effects and it is still widely used in Georgia and Russia [25]. Nevertheless, in Western countries it is still considered an unapproved treatment method. In Poland, the Institute of Immunology and Experimental Therapy, Polish Academy of Sciences, in Wroclaw collected vast experience providing phage therapy as experimental treatment to patients with various recalcitrant bacterial infections, including sinusitis. Application of bacteriophages was shown to be safe and it was associated with clinical improvement in 77%–83% of patients with sinusitis [49,50].

Recently, a phase 1 clinical trial of a phage cocktail against *S. aureus* in CRS patients was conducted in Australia [51,52]. The product was self-administered by nine post-ESS patients, who had failed all other medical therapies. Out of 25 patients who entered the study and did not declare withdrawal, 9 (36%) harbored bacteria susceptible to the cocktail. Overall, the sensitivity of *S. aureus* isolates to the preparation was 80%. The treatment was well tolerated and caused no serious adverse events. It resulted in reduction of *S. aureus* growth or eradication of the infection, nevertheless, it did not cause significant alleviation of symptoms. As discussed earlier, symptomatic improvement could probably be more apparent in cases of recent acute exacerbation than in patients with longstanding and recalcitrant baseline symptoms of CRS.

To assess the potential applicability of phage therapy in the population of patients with post-ESS AECRS, we decided on a different approach than in the in vitro studies described above. Instead of selecting one bacterial species for phage typing, we prospectively tested the phage sensitivity of all bacterial strains isolated from 50 consecutive patients who presented with relevant signs and symptoms. The results seem to be encouraging.

Generally, the products used for phage therapy can be either patient-tailored or preformed. The first approach requires phage typing of bacteria isolated from the patient and application of a custom-produced bacteriophage preparation. The second method utilizes broad spectrum cocktails that contain several bacteriophages active against various strains. In our study group, individualized therapy with phages selected from the collection could potentially be used in 60% of patients. Thirty-eight percent of the patients could potentially be treated solely with bacteriophages, as they carried only phage-sensitive pathogens. In 22% of patients, bacteriophages could be used as an adjunct to antibiotics, as both phage-sensitive and phage-resistant pathogens occurred together. Ready phage mixtures against *S. aureus* and *P. aeruginosa* would be suitable for 40% of patients. The most important findings of our study concern the phage susceptibility of antibiotic-resistant strains in the population of patients with AECRS. Eighty-one percent of these strains were sensitive to phages from the collection.

### 3.3. Limitations of the Study

- Culture provides limited information compared to molecular methods that would allow for more profound analysis of the microbiota in AECRS [13]. Culture-dependent techniques were utilized in this study because they were required for phage typing, which was the essential part of the project.
- The current study did not include identification of anaerobes; further research is required to address this problem.
- Phage susceptibility testing was limited to the contents of the collection available for our study. However, the collection is still being developed and there is a possibility of phage isolation on demand.
- Further research is required to test the phage sensitivity of bacteria in biofilms and in polymicrobial communities.

## 4. Materials and Methods 

The study protocol was approved by the Jagiellonian University Medical College Bioethics Committee (registry no: 1072.6120.208.2017). Written informed consent was obtained from all participants prior to enrollment.

### 4.1. Patient Recruitment

Adult patients with AECRS were recruited from the outpatient clinic of the Department of Otolaryngology, Jagiellonian University Medical College in Krakow between May 2018 and May 2019. The diagnosis of CRS was made based on the diagnostic criteria of the EPOS guidelines [22]. All of the participants had undergone endoscopic sinus surgery (post-ESS patients). AECRS was diagnosed according to the criteria suggested by Orlandi et al. [17] (worsening of symptoms and purulence on endoscopic examination, which usually was accompanied by crusting, hyperemic, and edematous mucosa and frequently polyp formation). If the history, symptoms, and endoscopic findings suggested a nonbacterial etiology of the exacerbation, such as allergy or viral infection, the patient was excluded from the study. Patients were also not enrolled if they had received any systemic or topical antibiotics in the week preceding sample collection or fulfilled the EPOS criteria of exclusion from general studies [22].

### 4.2. Specimen Collection

The swabs were collected directly from the infected sinuses under endoscopic guidance (in most postoperative patients the cavities were readily accessible for sample collection). As recommended by Nadel et al. [53], the swabs were placed directly into the pathological secretions. Contamination from the nares was carefully avoided and any swabs that could have come into contact with the nasal vestibule were discarded. The specimens were delivered to the laboratory as soon as possible after collection. Specimens for bacterial culture were transported at 4 °C in Amies medium.

### 4.3. Bacterial Culture and Identification

The swabs were inoculated on the Columbia agar with 5% sheep blood (OXOID) for Gram-positive aerobic cocci and on the selective MacConkey agar (OXOID) for the isolation of Gram-negative bacilli and the chocolate base agar with bacitracin (OXOID) for the isolation of *Haemophilus*. After 18 to 24 hours of incubation at 37 °C in the atmosphere containing 5% CO_2_, bacterial colonies were isolated and identified. Microorganisms were identified using a BD Phoenix (Becton Dickinson) automated microbiology system and appropriate test kits dedicated for Gram-negative and Gram-positive bacteria. *Haemophilus* rods were identified with discs containing bacitracin and factors V and X on Müller–Hinton agar plates (OXOID) by incubating a McFarland 0.5 suspension with paper discs for 24 hours at 37 °C with access to CO_2_.

### 4.4. Determination of Antibiotic Resistance

Bacterial isolates were tested according to EUCAST (The European Committee on Antimicrobial Susceptibility Testing) version 6.0 [54], using disc diffusion methodology on Müller–Hinton agar plates (OXOID). Clinical breakpoints for bacteria were interpreted according to EUCAST v. 8.0. [55,56]. 

### 4.5. Phage Typing—Spot Test

The bacteriophages used for phage typing belonged to the collection of Biophage Pharma S.A. and included phages specific for: *S. aureus, P. aeruginosa, E. coli, A. baumannii,* and *K. pneumoniae* (used also for typing of *K. oxytoca)*, as well as two phage cocktails specific for *S. aureus* and *P. aeruginosa* (each containing four selected phages) [57,58]. The cocktail consisting of *S. aureus* phages was prepared with the phages Puł/14/14256, Kr/6/1934, W/5/14256, and Kos/10/22119 at a concentration of 10^8^ PFU/mL per phage. The electron microscopy scan showed that all bacteriophages belong to the *Myoviridae* family. The cocktail consisting of *P. aeruginosa* phages was prepared with the phages Kos/4/1815, Ku/89/1815, Jar/51/2117, and P/53/2117. The cocktail was composed of phages mixed at the same number of PFU per milliliter (10^8^ PFU/mL). The electron microscopy scan showed that the phage Jar/51/2117 belongs to the *Podoviridae* family, and the three remaining phages belong to the *Myoviridae* family.

Bacteriophages specific for other species isolated from the patients were not available in the collection.

A spot test was used as a method for determination of the bacteriophage’s host range in the bacterial collection [59,60,61]. The tested bacterial strains were grown in NZCYM broth (to the turbidity 0.5 McFarland). Three milliliters of molten soft agar (0.7%) was mixed with 100 µL of the bacterial cells and this mixture was then overlaid onto the surface of solidified basal NZCYM agar (1.5%). Ten microliters (about 1.0 × 10^8^ PFU/mL) of a phage suspension was spotted onto bacterial lawn, which was then incubated overnight at 37 °C. Bacterial sensitivity to bacteriophage was established by bacterial lysis at the spot where the phage was deposited. Each test was repeated two times. According to the degrees of clarity, the spots were differentiated into following categories:

- n—no clearing—no bacterial lysis in the spot;
- p—a few single plaques in the spot;
- o3—turbid spot—very weak bacterial lysis in the spot;
- o2—medium turbid spot—weak bacterial lysis in the spot;
- o1—almost clear spot—very weak bacterial growth in the spot;
- c—completely clear spot—complete bacterial lysis in the spot.

Bacterial strains were described as phage-sensitive if the typing results fell into the categories c, o1, or o2.

## 5. Conclusions

In this study we have demonstrated that *S. aureus*, *H. influenzae*, *P. aeruginosa,* and *Enterobacteriaceae* are the species most commonly cultured from the patients with post-ESS AECRS. Disturbingly, in 28% of isolated species we detected mechanisms of antibiotic resistance. Our observations suggest that due to different microbiology and frequent resistance of the pathogens to amoxicillin, treatment of AECRS should not follow the recommendations for ABRS. The lowest rates of resistance were observed for fluoroquinolones and aminoglycosides. Fortunately, 59% of the pathogens isolated from the patients were sensitive to phages, including 81% of the antibiotic-resistant bacteria. Phage preparations that could be used instead of, or as an adjunct to, antibiotics are currently available for at least 60% of patients with AECRS. Preformed phage cocktails that could potentially be introduced without previous typing could be effective in 63% of patients with *S. aureus* infections and 40% of patients with *P. aeruginosa* infections. Definitely, further expansion of the phage collection is necessary to address infections caused by less common pathogens. 

## Figures and Tables

**Table 1:** Characteristics of the study group. CRS—chronic rhinosinusitis; ESS—endoscopic sinus surgery.

Gender	Male 23 (46%) Female 27 (54%)
Age	25–80 (mean 51)
Nasal polyps	45 (90%)
Comorbidities	
Asthma	27 (54%)
Allergy (to pollen, dust mites, etc.)	19 (38%)
Aspirin-exacerbated respiratory disease	10 (20%)
Gastroesophageal reflux	8 (16%)
History of CRS (years)	1.5–45 (median 10)
History of recurrent exacerbations and repeated antibiotic treatment	31 (62%)
Number of prior ESS procedures	1–5 (median 1.5)
Time since the last ESS (months)	1–96 (median 11)
Lund–Mackay computed tomography staging score [29] prior to surgery (total 0–24)	6–24 (median 15)
Modified Lund–Kennedy endoscopic score [30] on enrollment (0–2 for polyps, edema, discharge on each side, total 0–12)	2–12 (mean 6)
